# Deep Learning in RNA Structure Studies

**DOI:** 10.3389/fmolb.2022.869601

**Published:** 2022-05-23

**Authors:** Haopeng Yu, Yiman Qi, Yiliang Ding

**Affiliations:** Department of Cell and Developmental Biology, John Innes Centre, Norwich Research Park, Norwich, United Kingdom

**Keywords:** deep learning, RNA secondary structure, RNA tertiary structure, RNA structure prediction, RNA G-quadruplex, RNA-protein interaction

## Abstract

Deep learning, or artificial neural networks, is a type of machine learning algorithm that can decipher underlying relationships from large volumes of data and has been successfully applied to solve structural biology questions, such as RNA structure. RNA can fold into complex RNA structures by forming hydrogen bonds, thereby playing an essential role in biological processes. While experimental effort has enabled resolving RNA structure at the genome-wide scale, deep learning has been more recently introduced for studying RNA structure and its functionality. Here, we discuss successful applications of deep learning to solve RNA problems, including predictions of RNA structures, non-canonical G-quadruplex, RNA-protein interactions and RNA switches. Following these cases, we give a general guide to deep learning for solving RNA structure problems.

## Introduction

As a data-driven algorithm, deep learning has shown promise with successful applications in biology, healthcare, and drug discovery (Schmidhuber, 2015; Angermueller et al., 2016; Goh et al., 2017; Ching et al., 2018). One of the most recent deep learning breakthroughs has been to predict protein structure. In past decades, researchers needed to spend months or even years solving a complex protein structure using experimental methods like nuclear magnetic resonance (NMR) or cryo-electron microscopy (Cryo-EM). Based on this hard-earned data, deep learning models such as Alphafold2 and RoseTTAFold can predict protein structures from amino acid sequences that are remarkably close to those determined experimentally (Baek et al., 2021; Jumper et al., 2021). Currently, the protein structure database based on AlphaFold2 predictions has provided nearly one million protein structure models, far exceeding the experimentally determined structures in previous decades (Varadi et al., 2022). These advances in deep learning methods for predicting protein structures infer their applicability for studying RNA structure.

As a key in the central dogma, RNA is essential for gene expression. RNAs fold into RNA secondary structures by base pairing, which can fold further to form RNA tertiary structures. This RNA folding is extremely important for achieving RNA’s diverse and complex biological functions (Mathews and Turner, 2006; Mortimer et al., 2014; Zhang and Ding, 2021). For instance, transfer RNAs usually have a cloverleaf secondary structure with an L-shaped tertiary structure which can fit into ribosomal P and A sites for the translation (Holley et al., 1965; Kim et al., 1974). While long noncoding RNAs (lncRNAs) regulate genomic functions through their specific RNA structures (Qian et al., 2019). Research into the exploits of individual RNA structures and their functional importance is ongoing, with more recent experimental efforts in probing RNA structures over tens of thousands of RNAs in one single experiment transforming the scale for such study. A dramatic increase in RNA structure data resources has laid the foundation for the application of deep learning algorithms in deciphering general features for predicting RNA structure and its functions.

In this review, we have compiled examples from previous research whereby deep learning methods were adopted to solve RNA structure-related problems. Firstly, we introduce the brief process of deep learning modelling through a G-quadruplex classification question. Secondly, we propose that the availability of high-throughput sequencing data has facilitated deep learning modelling of RNA structure-related problems. Next, the experience of deep learning architecture design is presented through the examples of RNA-protein binding prediction and toehold-switch prediction models. Subsequently, we introduce classification and regression in supervised learning through RNA secondary structure prediction and RNA tertiary structure scoring. Lastly, we present several solutions for the interpretation of deep learning models as mentioned in studies. Our review provides an overview of deep learning modelling approaches from the perspective of RNA structure-related research, before providing suggestions for future efforts to address more questions in RNA structure by deep learning.

## The Basis of Deep Learning Modelling

Deep learning is a machine learning technique, capable of learning abstract features from high-dimensional data through multiple processing layers (LeCun et al., 2015). Imagine that we propose to build a model to determine whether a guanine-rich (G-rich) DNA or RNA sequence has the potential to fold into a G-quadruplex structure (GQS, a tertiary structure motif that is folded *via* Hoogsteen hydrogen-bonded guanines) (Bochman et al., 2012; Kwok et al., 2016a). In traditional modelling, the most important step is called “feature extraction”. For example, to build this GQS classification or scoring model, certain features need to be extracted based on previous knowledge, such as the number of adjacent guanines (“GG” or “GGG”), the length of the loops, the presence of bulge (like “GGAG”), whether the loop contains cytosine and the probability for the competition of adjacent canonical DNA or RNA secondary structure. However, if there are still features or non-linear combinations of features that are not considered, the model may struggle to achieve highly accurate predictions.

**FIGURE 1:**
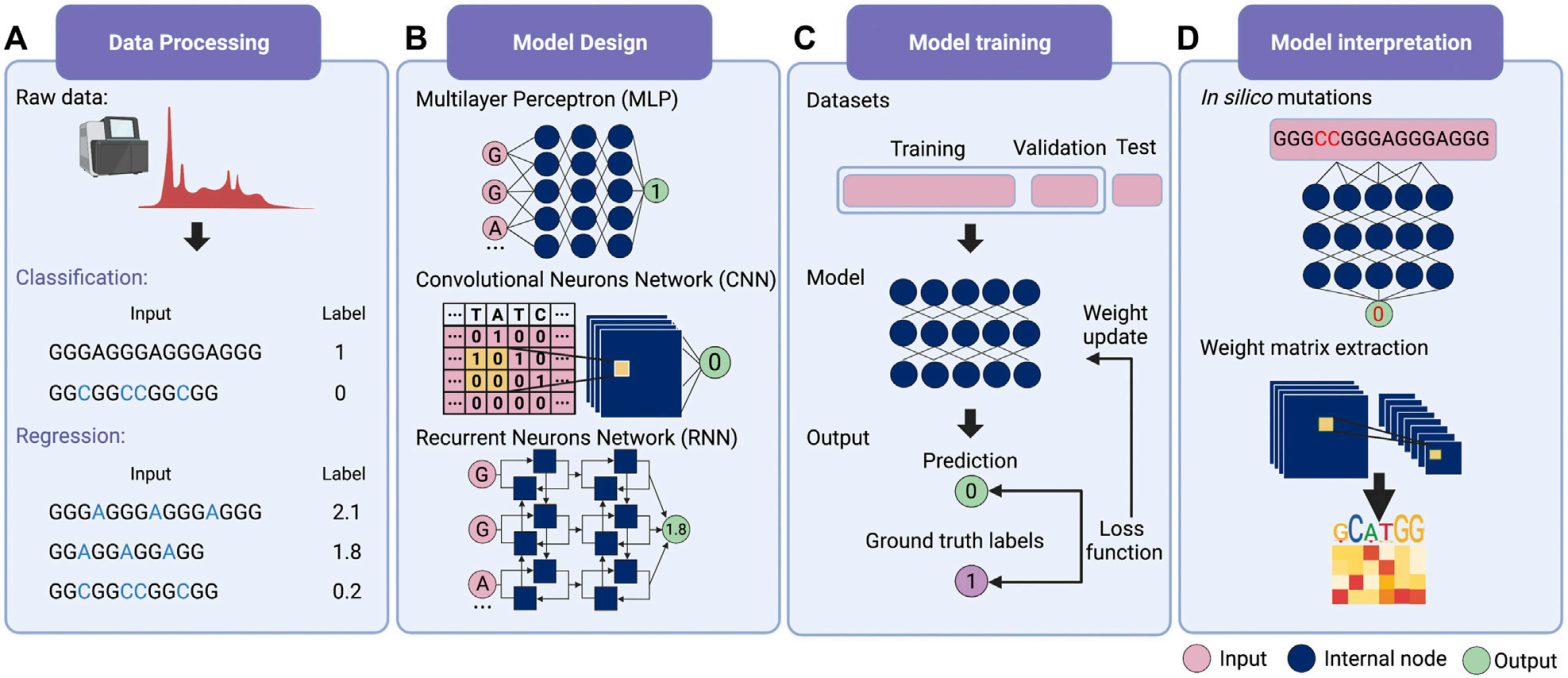
Schematic overview of deep learning workflow. **(A)** Data processing. Supervised learning requires explicit labelling of the data, including class numbers in classification questions and values in regression questions. **(B)** Model design. Multilayer perceptron (MLP), convolutional neural network (CNN) and recurrent neural network (RNN) are the three main families of deep learning architecture. Typically, deep learning models assemble different architectures based on data structures. **(C)** Model training. The total training data is first divided into the training set, the validation set and the test set. Then the input data is passed into the model to obtain the predicted values. The loss function is applied to evaluate the difference between the predicted and the true values, whereby the model weights are updated. **(D)** Model interpretation. Features’ importance can be obtained by *in silico* mutations. For the CNN model, the features can also be evaluated by extracting the weight matrix of the filter.

For deep learning, it is possible for modeling without feature extraction (Figure 1A). This particular question can be considered a bi-classification problem, i.e., classifying G-rich sequences into GQS or non-GQS classes. Instead of feature extraction, we can simply input the entire G-rich sequences into the model. We first need to prepare a large number of GQS or non-GQS sequences with clear classification labels, for example, GQS as “1” and non-GQS as “0”. After designing a deep learning model, the training process begins. The GQS and non-GQS sequences are fed into the model as inputs and their model-estimated classifications as outputs. Ideally, the classification estimated by the model should be as close to the true class as possible, but usually not in the initial training. Therefore, we need to set an “objective function” (also known as the “loss function”) for evaluating the error of the estimated classification from the true classification (Figure 1C). The model then updates its trainable parameters to reduce the error. Typically, deep learning models may have millions of trainable parameters, called weights. The model will calculate a gradient for each weight and determine the adjustment direction to reduce the error (known as “gradient descent”). Through continuous iterations corresponding to the constant updating of the weights, the classification predicted by the model progressively approaches the true classification (Figure 1C). Ultimately, a powerful deep learning model is derived for predicting the foldability of the G-rich sequence.

Several deep learning models are available for GQS classification prediction (Table 1). G4NN was trained using the MLP model on 149 experimentally identified RNA GQSs and 179 non-RNA GQSs from the G4RNA database, and the performance outperformed the scoring matrix-based RG4 prediction model (Garant et al., 2015, 2017). “PENGUINN”, adopted a sequence as input and a prediction classification score as output (Klimentova et al., 2020). In addition, it has a higher area under the precision-recall curve value (AUC) than methods based on regular expressions and scoring matrices (Klimentova et al., 2020). The “G4detector” introduces RNA structure information to improve GQS prediction (Barshai et al., 2021) and the “DeepG4” was trained on *in vivo* G4 data (G4 ChIP-seq) and identified key DNA motifs associated with GQS region activity (Rocher et al., 2021). “PENGUINN”, “G4detector” and “DeepG4” have been applied to DNA GQS structure prediction at the genome-wide level, further deep learning models based on rG4-seq and SHALiPE-Seq datasets for RNA GQS prediction at the transcriptome-wide level can be expected to emerge in the future as well.

**TABLE 1:** Deep learning-based models in RNA structure.

Function	Name	Model	Method highlights	Link
RNA secondary structure prediction	SPOT-RNA Singh et al. (2019)	ResNet, LSTM	The model was first trained with a large volume of RNA secondary structures, then trained again using a transfer learning strategy on a small number of validated RNA structures	https://github.com/jaswindersingh2/SPOT-RNA/
	CDPfold Zhang et al. (2019)	CNN, MLP	Predicts the pairing probability matrix of RNA structures and applies dynamic programming methods to generate RNA structures	https://github.com/zhangch994/CDPfold
	DMfold Wang et al. (2019)	Bi-LSTM	Predicts the pairing probability matrix of RNA structures and applies IBPMP methods to generate RNA structures	https://github.com/linyuwangPHD/RNA-Secondary-Structure-Database
	Calonaci et al. (2020)	CNN, MLP	Integrates RNA thermodynamic method, chemical probing data and co-evolutionary information into the model	https://github.com/bussilab/shape-dca-data
	Willmott et al. (2020)	Bi-LSTM	Generates synthetic SHAPE data for RNA structure prediction	https://github.com/dwillmott/rna-state-inf
	MXfold2 (Sato et al., 2021)	CNN, Bi-LSTM	Four types of the folding score were calculated for each nucleotide pair	https://github.com/keio-bioinformatics/mxfold2
	Ufold (Fu et al., 2022)	FCN	The input is instead of RNA sequences but a matrix of 16 possible pairings and pairing features for each base pair	https://github.com/uci-cbcl/UFold
RNA tertiary structure scoring	ARES Townshend et al. (2021)	MLP	The model first generated many potential RNA structures by sampling and predicting their different score from the true structure, thus overcoming the problem of insufficient RNA tertiary structures	http://drorlab.stanford.edu/ares.html
G-quadruplexes structure prediction	G4NN Garant et al. (2017)	MLP	The model is trained on experimentally validated RNA GQSs and provides a stability score for RNA GQSs	http://scottgroup.med.usherbrooke.ca/G4RNA_screener/
	PENGUINN Klimentova et al. (2020)	CNN	Robustness to unbalanced data sets and easy-to-use web interface	https://ml-bioinfo-ceitec.github.io/penguinn/
	G4detector Barshai et al. (2021)	CNN	Introduces RNA secondary structure information into the model to improve G4 prediction	https://github.com/OrensteinLab/G4detector
	DeepG4 Rocher et al. (2021)	CNN, MLP	The model is trained on *in vivo* G4 data (G4 ChIP-seq)	https://github.com/morphos30/DeepG4
RNA structure-mediated protein interactions prediction	iDeepS Pan et al. (2018)	CNN, Bi-LSTM	Combines RNA sequence and RNA structure as input during model training	https://github.com/xypan1232/iDeepS
	PrismNet Sun et al. (2021)	CNN, ResNet, SE network	Integrates experimental *in vivo* RNA structure data during model training	https://github.com/kuixu/PrismNet
RNA structure-mediated regulatory elements prediction	Angenent-Mari et al. (2020)	MLP	Comparably, this outperforming model was achieved by using RNA sequences directly as input data, rather than extracted features	https://github.com/lrsoenksen/CL_RNA_SynthBio

## Data First

In addition to improvements in computer power and high capacity models, the success of deep learning is largely attributable to the availability of large-scale annotated data (Sun et al., 2017). Fortunately, evolving technologies have provided researchers with a wealth of novel tools, especially high-throughput sequencing (HTS), allowing for an explosion of biological data (Mahmud et al., 2021). For example, several HTS methods were applied to detect GQS at both DNA and RNA levels (G4-seq and G4 ChIP-seq for DNA, rG4-seq and SHALiPE-seq for RNA), and thus induced the creation of deep learning models such as “PENGUINN”, “G4detector”, and “DeepG4” (Chambers et al., 2015; Kwok et al., 2016b; Hänsel-Hertsch et al., 2016; Klimentova et al., 2020; Yang et al., 2020; Barshai et al., 2021; Rocher et al., 2021).

In RNA structure detection, recent high-throughput *in vitro* and *in vivo* RNA structure chemical probing methods can achieve nucleotide-resolution RNA structure information over tens of thousands of RNAs (RNA structure information of over 50 million nucleotides) in one single experiment, transforming the scale of RNA structure study to an unprecedented level (Ding et al., 2014; Rouskin et al., 2014; Spitale et al., 2015; Yu et al., 2020). These methods utilise chemicals such as dimethyl sulfate (DMS) and SHAPE (Selective 2′-Hydroxyl Acylation analysed by Primer Extension) that determine the single-strandedness of RNA nucleotides. These large volumes of high-throughput sequencing data provide the potential for improving the accuracy of RNA structure prediction. Calonaci et al. established a compound deep learning model to combine multiple channels of RNA sequence information, chemical probing data (single-strandedness information) alongside direct coupling information (derived from co-evolutionary data) to build the thermodynamic prediction method (Calonaci et al., 2020). Further penalties derived from known RNA structures from the Protein Data Bank (PDB) database were applied as perturbations to the thermodynamic prediction (Calonaci et al., 2020).

RNA-protein interactions are integral to core biological processes, ranging from transcriptional and post-transcriptional regulation (Castello et al., 2012). With the increase of high throughput data on RNA binding protein binding sites, like CLIP-Seq, deep learning methods were developed as a consequence to better predict RNA-protein interactions (Table 1). Notably, RNA-binding proteins (RBP) recognise specific RNA sequences and specific RNA structure features (Mortimer et al., 2014; Lewis et al., 2017; Zhang and Ding, 2021). For example, PrismNet (Protein-RNA Interaction by Structure informed Modeling using deep neural NETwork) was constructed by integrating RNA sequence, RBP binding sites, and *in vivo* RNA structure information to predict the impact on RNA-protein interaction by one single-nucleotide variant (SNV) that disrupts RNA structures (Sun et al., 2021). This trained model can also predict the dynamics of the interaction between RNA structural mutations and RNA binding protein from a huge volume of disease-associated mutations; such large-scale assessments are impossible for experimental methods (Sun et al., 2021).

## Design of Deep Learning Architectures

The deep learning model is not trained to fit existing data. Instead, it is required to predict independent, unknown data, i.e., generalisation. If the model only has a good fitting on the training set, it is called “overfitting”, that is, the model may have a large bias in predicting non-training set data. For this purpose, the input data is first normalised and thoroughly shuffled to ensure that the samples have the same distribution. Then, the data set is usually randomly divided into three parts: training set, validation set and test set (Figure 1C) (Goodfellow et al., 2016). The training set is utilised to fit the model and the validation set for unbiased evaluation of an optimal model. Furthermore, a set of independent, unused samples is required for testing generalisability, and this is the test set. Typically, the ratio of the training set is maximised during model training. By way of illustration, for the ratio of training, validation and test sets in the prediction of RNA secondary structure, SPOT-RNA and E2Efold were established with the ratio of 8:1:1, while CDPfold adopted the ratio of 7:2:1 (Singh et al., 2019; Zhang et al., 2019; Chen et al., 2020). In addition, it is feasible to divide the data into k parts and use 1 of these parts as the test set and the remaining k-1 parts as the training set respectively to obtain the average performance of the model on this data set (also known as the “k-fold cross-validation”) (Goodfellow et al., 2016).

The next step is to consider the design of deep learning architectures. There are mainly three families of deep learning architectures: feed-forward neurons network, convolutional neurons network (CNN) and recurrent neurons network (RNN) (Zou et al., 2019) (Figure 1B). The feed-forward network is the basic architecture and is also known as a multilayer perceptron (MLP) when each layer is a fully connected layer. CNN can receive input data in matrix form and scan the matrix by introducing ‘filters' to calculate a sum of local weights so that local features can be captured (Krizhevsky et al., 2012). RNN was originally designed for sequential and time-series data and was enabled to “remember” the previous state of the series data to influence the current input and output (Zaremba et al., 2015).

Typically a deep learning model connects one or more architectures like “building blocks”. Then the entire model works like a pipeline, moving the input data ‘through' the different architectures, layer by layer, to obtain the predicted values (Figure 1C). For example, iDeepS, a deep learning-based method, combined a CNN and a bidirectional LSTM (Bi-LSTM, is a special kind of RNN) to predict RNA-protein binding preferences. The CNNs were first applied to determine the abstract features of both RNA sequence and *in silico* predicted RNA structure. The close relationship between RNA sequence and structure was then captured by Bi-LSTM for an estimate of possible long-range dependencies (Pan et al., 2018). The deep-learned weighted representations were then fed into a classification layer for predicting RNA binding protein (RBP) sites (Pan et al., 2018). Prediction values derived from iDeepS were verified by CLIP-seq by combining UV cross-linking with immunoprecipitation for analysing protein interactions with RNAs (Pan et al., 2018). This method outperforms the sequence-only prediction methods, indicating the importance of RNA structure features in RNA-protein binding.

In research on toehold switch prediction by deep learning, Angenent-Mari et al. adopted different deep learning models and performed a comparison. The toehold switch is the type of RNA switch that controls downstream translation by its hairpin structure and programmable trans-RNA sequence (Green et al., 2014). Much effort in RNA synthetic biology has attempted to improve the prediction of toehold switch functionality based on thermodynamic modelling and limited datasets. Angenent-Mari et al. expanded toehold switch datasets from <1,000 to the 10^5^ level by high-throughput DNA synthesis and sequencing pipeline and then presented different architectures for deep learning models to extract the desired sequence features (Angenent-Mari et al., 2020). The three-layer deep learning MLP model based on these datasets has a ten-fold improvement on the linear regression model. Then, the model with only RNA sequences as input and the model with 30 rational thermodynamic features as input were compared. Based on the results, the sequence-only model doubled the performance of the feature-extraction model, presumably as the 30 features were not fully inclusive of all the information hidden in the sequences (Angenent-Mari et al., 2020). Notably, the model with inputs of both thermodynamic features and RNA sequences did not significantly outperform the sequence-only model (Angenent-Mari et al., 2020). Interestingly, the more complex model architectures, CNN and LSTM, were also utilised for training the same toehold-switch datasets but did not outperform the MLP model (Angenent-Mari et al., 2020).

## Supervised Learning in RNA Structure Prediction

The most common form of deep learning is the supervised learning (LeCun et al., 2015). In supervised learning, the goal of the model is to enable the predictions to be as close as possible to the labels, both discontinuous labels (classification) and continuous labels (regression). The model mentioned above for predicting whether a G-rich sequence can form a GQS is a typical classification question. Also, RNA secondary structure prediction can be achieved by classifying each base pair’s status (pair or not) (Table 1).

For example, Singh et al. developed an RNA secondary structure prediction model, SPOT-RNA, with RNA sequence as input and the pairing classification status of each potential base pair as output (an L × L matrix, L is the length of RNA sequence) (Singh et al., 2019). SPOT-RNA was developed to train an ensemble of ultra-deep hybrid networks of Residual Neural Network (ResNet) and LSTM with 13,419 RNA structures in the bpRNA database (Danaee et al., 2018; Singh et al., 2019). This large model was then trained on a small dataset of 217 validated high-resolution RNA structures. This transfer learning strategy was shown to improve prediction performance by 13% over the next-best model in direct RNA secondary structure prediction. Another software, E2Efold, adopts a deep learning approach to obtain the bi-classification scoring matrix of base pairs from the input RNA sequences, and then constrains the output space by an unrolled algorithm-based Post-Processing Network to achieve an end-to-end RNA structure prediction model (Chen et al., 2020). In addition to bi-classification, CDPfold adopted a CNN model to predict RNA pairing probability matrices of three labels ("(", ")" and ".") and further combined a dynamic programming algorithm to generate optimal RNA structures (Zhang et al., 2019). DMfold supports the prediction of seven RNA secondary structure dot-bracket symbols for each base, thus incorporating knowledge of the prediction of RNA pseudoknot structure (Wang et al., 2019). Non-classification deep learning algorithms have also been applied to RNA secondary structure prediction models. MXfold2 was trained with thermodynamic regularisation to ensure the predicted four types (helix stacking, helix opening, helix closing and unpaired region) of folding scores are close to the calculated free energy (Sato et al., 2021). Instead of inputting the RNA sequence directly, another model, Ufold, inputs an ‘image' of the RNA sequence, a matrix of all possible base pairings (canonical and non-canonical base pairing) and pairing features (Fu et al., 2022). By employing the Fully Convolutional Networks (FCNs), Ufold transformed this RNA sequence ‘image' into base-pairing probabilities for predicting RNA secondary structures (Fu et al., 2022).

In supervised learning, if the labels are continuous values, it becomes a regression question. Recently, the model for scoring RNA tertiary structures, ARES, is an example of a deep learning regression model developed with a small amount of training data (Table 1). In comparison with the ∼100,000 unique protein structures, there are only 3,335 non-redundant RNA 3D structures (from “the Representative Sets of RNA 3D Structures database”, version 3.225), whereby most RNA tertiary structures are RNA fragments under 100bp (Leontis and Zirbel, 2012). This was mainly due to limitations of experimental methods to resolve RNA structures that are largely unstable, very dynamic, and have high plasticity. Unlike protein structure predictions using Alphafold2 and RoseTTAFold based on extensive data resources, only limited known RNA tertiary structures were available for RNA structure prediction. Townshend et al. trained a novel RNA tertiary structure scoring model, the Atomic Rotationally Equivariant Scorer (ARES), by 18 known RNA tertiary structures published between 1994 and 2006 (Das and Baker, 2007; Townshend et al., 2021). Unlike a direct prediction of RNA tertiary structure (sequence as input and tertiary structure as output), researchers first generated 1000 RNA tertiary structural models using the Rosetta FARFAR2 sampling method. Each derived RNA structural model was then assessed for the differences between each of its atom’s positions and the corresponding atom of the known RNA structures, that is, the true root mean square deviation (RMSD) (Townshend et al., 2021). Next, the deep learning model was released, with the input being the atoms' features and the output being the RMSD for each generated RNA tertiary structure model. The ARES model is a sequential model containing an atomic embedding layer, a self-interaction layer, an equivariant convolution layer, and Multilayer Perceptron (MLP) with exponential linear units (Hinton and Salakhutdinov, 2006; Clevert et al., 2016; Thomas et al., 2018). As an RNA tertiary structure scoring model, ARES significantly outperforms the other scoring functions and models despite using a limited number of known RNA structures (Townshend et al., 2021).

## Interpreting Deep Learning Models

A deep learning model is typically thought of as a “black box” containing millions of weights that predict input data as output values. But for researchers in biology, the biological features that the model learns and the biological questions it can explain are more important than just predictions. Contrary to standard statistical models and machine learning methods based on features extraction, deep learning models are challenging to interpret (Zou et al., 2019).

The most straightforward interpretation means is to perform an *in silico* mutations by algorithm (Figure 1D) (Zou et al., 2019). This approach requires large-scale *in silico* mutations of the input data followed by re-prediction with the model to assess the impact of changes in the input on the output. For example, for a bi-classification model for GQS prediction, it is possible to simulate base-by-base mutations to alter the input sequence and predict its classification, thus evaluating which nucleotides affect GQS folding. During translation, ribosomes are known to actively unwind the RNA structure where a complex interaction between the ribosome and RNA structure occurs. DeepDRU is a deep learning model for predicting the unwinding state of RNA structures *in vivo* (Yu et al., 2019). This research demonstrated that ribosome occupancy has a greater impact on the unwinding degree of RNA structure *in vivo* than the sequence itself by simulating mutations of a feature while the rest of the features are fixed (Yu et al., 2019).

For interpreting CNN models, the convolutional filters in the model can be visualised as heat maps or position weight matrices to extract the high-level patterns learned (Figure 1D). In the model for RBP prediction, DeepBind and iDeep adopted this approach to extract the parameter matrix of the filters from the first-layer convolutional network to identify the RBP binding motifs (Alipanahi et al., 2015; Pan and Shen, 2017). Another RPB prediction model, PrismNet, incorporates “SmoothGrad” to visualise enhanced saliency maps for identifying the high attention regions of RNA sequence leading to the extraction of RBP binding motifs (Sun et al., 2021). Notably, the interpretation of the model is a purely computational simulation based on a model with well-generalised properties, and the proof of the relevant conclusions may require subsequent experimental validation.

## Discussion

The emergence of data-driven deep learning approaches integrates technological innovation, “Big Data” exploitation, and huge computational power to significantly transform the scale for studying RNA structures and their functions. We introduce the basic concepts of deep learning, the importance of data volumn, supervised learning, design of deep learning architectures and model interpretation by reviewing recent deep learning applications in deciphering different aspects of RNA structure studies and highlighting those that demonstrate the best potential for future development.

Although deep learning has shown promise for application in the RNA structure field, there are still some issues that need to be addressed. Firstly overfitting is presently the major risk to deep learning models, especially when faced with limited data size. Advances in technology have led to the development of multi-layered, high-capacity models that can be applied to obtain features for more complex data structures. However, simultaneously, the risk of overfitting arises. In a recent study of RNA secondary structure prediction, it was suggested that E2Efold may suffer from overfitting and is therefore not suitable for predicting broader datasets (Sato et al., 2021; Fu et al., 2022). Hence it is far more important to develop highly available experimental datasets in the future than to adopt models with higher levels of capacity. Another challenge is to give a suitable biological interpretation to the purely computationally generated models and the relevant patterns learnt and how to apply deep learning models to complement human experience for functional RNA structure design. With sufficient data, more complex models always imply better performance, but at the same time become difficult to interpret. Typically, the complexity of a model is inversely proportional to the interpretability. In contrast to deep learning, ‘non-deep' algorithms, such as decision tree algorithms, can have good interpretability by obtaining the weights of individual features. Therefore, we need to make a trade-off between model complexity and interpretability according to the specific objectives.

Encouragingly, with the dramatic increase of high throughput RNA structure data generated from different organisms under diverse conditions, deep learning will be increasingly appreciated by RNA structure researchers and be progressively used to deduce RNA structure information and associated functionality. As the rise of available deep learning models increases, it will become progressively easier for researchers to apply deep learning in their routine data analysis for studying RNA structures.
